# Incorporating variability in simulations of seasonally forced phenology using integral projection models

**DOI:** 10.1002/ece3.3590

**Published:** 2017-11-26

**Authors:** Devin W. Goodsman, Brian H. Aukema, Nate G. McDowell, Richard S. Middleton, Chonggang Xu

**Affiliations:** ^1^ Earth and Environmental Science Division Los Alamos National Laboratory Los Alamos NM USA; ^2^ Department of Entomology University of Minnesota St Paul MN USA; ^3^ Pacific Northwest National Laboratory Richland WA USA

**Keywords:** bark beetle, individual‐based model, insect, phenology model

## Abstract

Phenology models are becoming increasingly important tools to accurately predict how climate change will impact the life histories of organisms. We propose a class of integral projection phenology models derived from stochastic individual‐based models of insect development and demography. Our derivation, which is based on the rate summation concept, produces integral projection models that capture the effect of phenotypic rate variability on insect phenology, but which are typically more computationally frugal than equivalent individual‐based phenology models. We demonstrate our approach using a temperature‐dependent model of the demography of the mountain pine beetle (*Dendroctonus ponderosae* Hopkins), an insect that kills mature pine trees. This work illustrates how a wide range of stochastic phenology models can be reformulated as integral projection models. Due to their computational efficiency, these integral projection models are suitable for deployment in large‐scale simulations, such as studies of altered pest distributions under climate change.

## 1 INTRODUCTION

Climate change is influencing the phenology of insects and plants with temperature‐dependent development rates (Bentz et al., 2010; Cleland, Chuine, Menzel, Mooney, & Schwartz, 2007; Régnière, St‐Amant, & Duval, 2012). Scientists have developed a range of temperature‐dependent phenology models to understand and predict how phenology will change under a warmer climate including degree‐day models (Tobin, Nagarkatti, Loeb, & Saunders, 2008), cohort‐based models (Logan, 1988), individual‐based models (Régnière, Bentz, Powell, & St‐Amant, 2015; Régnière & Powell, 2013; Régnière, St‐Amant, et al., 2012), and models based on partial differential equations such as the McKendrick–von Foerster equation and its extensions (von Foerster, 1959; Gilbert, Powell, Logan, & Bentz, 2004; McKendrick, 1926). Regardless of the modeling approach, most models of seasonally forced phenology rely on rate summation (Logan, 1988) to compute the level of maturation that has accumulated as a function of a series of temperatures experienced by organisms. In rate summation, the development rate multiplied by the time interval (approximate level of development accrued in the time interval) at each time step is cumulatively summed over a time series of varying temperatures experienced by an organism. Once a certain level of development has accumulated, the organism matures into the next stage of its life cycle and the timing of development completion constitutes the phenological prediction or observation.

Popular degree‐day‐based phenology models that are commonly used to model crop and crop pest phenology (Herms, 2004; Sharratt, Sheaffer, & Baker, 1989) are a subtype of rate summation models in which daily temperatures above the lower temperature threshold are summed over a relevant number of days and specific phenological events are predicted to occur at prescribed cumulative degree‐day milestones (Dennis, Kemp, & Beckwith, 1986; Kemp, Dennis, & Beckwith, 1986; Pruess, 1983). More general rate summation models involve development rates that vary nonlinearly with temperature (Logan, 1988; Régnière, Powell, Bentz, & Nealis, 2012; Schoolfield, Sharpe, & Magnuson, 1981; Sharpe & DeMichele, 1977; Wagner, Wu, Sharpe, & Coulson, 1984). Phenology models based on the rate summation concept are primarily used for insect phenology modeling (Herms, 2004; Logan, 1988; Régnière, Powell, et al., 2012; Régnière, St‐Amant, et al., 2012), but have also been used to model the phenology of plants (Ghersa & Holt, 1995; Osawa, Shoemaker, & Stedinger, 1983; Sharratt et al., 1989) and parasitic nematodes (Molnár, Kutz, Hoar, & Dobson, 2013).

Stochastic rate variability can have important consequences in rate summation models that are distinct from the effects of rate variability due to seasonal forcing (Gilbert et al., 2004; Régnière et al., 2015; Régnière, St‐Amant, et al., 2012). Stochastic rate variability can originate, for example, from intraspecific phenotypic variation that generates apparent randomness in observed demographic parameters (Régnière & Powell, 2013). As a result, demographic parameters or populations themselves may be better described by distributions than by scalar values. A continuous‐time stage‐structured model, the Mckendric–von Foerster equation (von Foerster, 1959; McKendrick, 1926), has been extended to accommodate a distributed aging rate and used to model the flight phenology of mountain pine beetles (Gilbert et al., 2004). Researchers have similarly incorporated the effects of rate variability and forcing by seasonal fluctuations within individual‐based stage‐ and age‐structured models (Régnière & Powell, 2013; Régnière et al., 2015; Régnière, St‐Amant, et al., 2012).

Although the effects of rate variability and seasonal forcing can be readily simulated using individual‐based methods (Régnière & Powell, 2013; Régnière et al., 2015; Régnière, St‐Amant, et al., 2012), the computational cost of simulation in individual‐based models restricts their use in large‐scale earth system models (Régnière et al., 2015). An alternate framework for simulating rate variability in seasonally forced age‐ and stage‐structured models is the cohort‐based approach (Focks, Daniels, Haile, & Keesling, 1995; Focks, Haile, Daniels, & Mount, 1993; Legros et al., 2011; Logan, 1988; Magori et al., 2009). In cohort‐based models, individuals born at varying times are binned into separate categories and the phenology of each cohort is tracked through time. Although binning into cohorts increases computational efficiency relative to individual‐based simulation, there are drawbacks to artificial categorization (binning) of individuals into discrete age or size classes. Accuracy drawbacks due to binning in cohort‐based models are analogous to those associated with dividing age into discrete categories in matrix models of demography (Ellner & Rees, 2006).

Accuracy issues due to binning can be minimized by replacing matrix or cohort‐based models with integral projection models (Ellner & Rees, 2006), which permit continuous age distributions. Integral projection models have been used to predict flowering phenology in plants while simultaneously modeling demography (Ellner & Rees, 2006). Due to its flexibility, the integral projection framework has been extended to include stochastic processes (Childs, Rees, Rose, Grubb, & Ellner, 2004; Ellner & Rees, 2007; Rees & Ellner, 2009; de Valpine, 2009), time‐varying parameters (Rees & Ellner, 2009), evolving traits (Coulson & Tuljapurkar, 2008; Coulson, Tuljapurkar, & Childs, 2010), and parameter variability (Plard, Gaillard, Coulson, & Tuljapurkar, 2016). Although integral projection models have been shown to be useful for modeling phenology (Ellner & Rees, 2006) and stochastic processes (Childs et al., 2004; Ellner & Rees, 2007; Rees & Ellner, 2009; de Valpine, 2009), as far as we know, no previous work has derived integral projection models starting from the stochastic rate summation process that underlies most phenology models.

In this study, we start from the rate summation concept and derive computationally efficient age‐structured integral projection models that incorporate stochastic rate variability while accommodating seasonal forcing. The mathematics underlying the derivation differ from the mathematics of traditional integral projection models but enable the simulation of the stochastic process that results in a distributed population with respect to a continuous physiological age variable in the presence of seasonal forcing. Linking age‐structured integral projection models of development in each life stage enables simulation of an organism's entire life cycle or multiple life cycles and results in a stage‐ and age‐structured integral projection model. Our approach realistically incorporates seasonal forcing using an underlying model that is well established in the phenology modeling community, while also accounting for the effects of rate variability.

**Figure 1:**
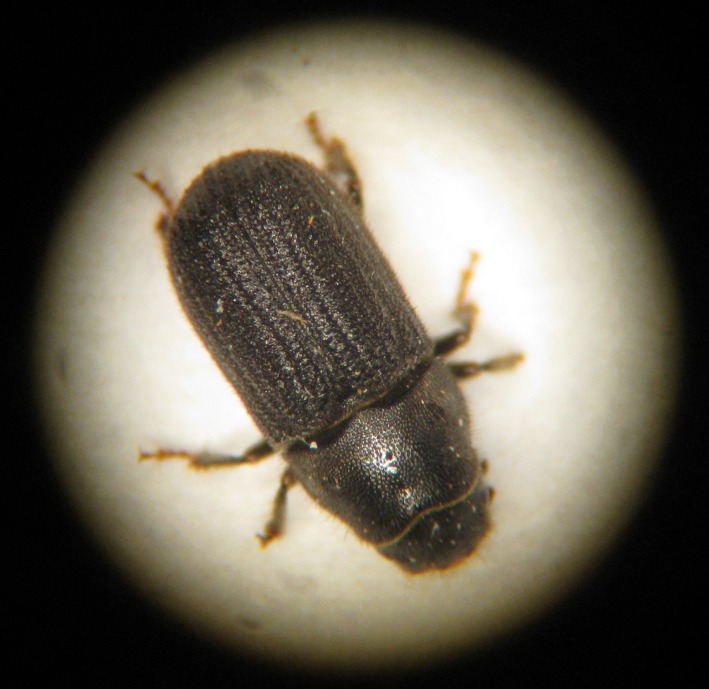
An adult mountain pine beetle (*Dendroctonus ponderosae* Hopkins) photographed under a light microscope. The adult mountain pine beetle is about the length of a grain of rice. Photograph credit: Devin W. Goodsman

We demonstrate the utility of stage‐ and age‐structured integral projection models for accommodating phenotypic variation in rate parameters using a temperature‐dependent model of mountain pine beetle (*Dendroctonus ponderosae* Hopkins) phenology and mortality. The mountain pine beetle (Figure 1) is a tree‐killing bark beetle that causes landscape‐wide mortality in mature pine forests when at outbreak levels (Bentz et al., 2010; Raffa et al., 2008). The mountain pine beetle system is ideal for demonstrating the advantages of our modeling framework as the phenology and demography of mountain pine beetles are strongly influenced by environmental temperatures (Safranyik & Carroll, 2006) and individual‐level variability in phenotypic traits has been quantified (Régnière & Powell, 2013; Régnière, Powell, et al., 2012).

## 2 MATERIALS AND METHODS

We begin by defining a simple integral projection model for age‐structured demography in an organism with a single life stage. We then demonstrate how integral projection models can be derived as solutions of stochastic rate summation models with seasonal forcing. We briefly describe how integral projection models derived from rate summation can be linked to form stage‐ and age‐structured integral projection models that incorporate temporally varying environment‐dependent mortality and birth. After describing an application of our integral projection modeling framework to model mountain pine beetle phenology, we conclude with description of our model validation against mountain pine beetle flight phenology data from Alberta, Canada.

### 2.1 Model derivation

#### 2.1.1 The integral projection model

Integral projection models represent the evolution of continuous variables such as age or size when time is iterated forward in discrete time steps. One can think of integral projection models as analogous to demographic matrix models in which the Leslie matrix (Leslie, 1945, 1948) for discrete age categories has been replaced with a projection kernel that represents the growth, mortality, and aging processes with age treated as a continuous variable (Ellner & Rees, 2006). A simple integral projection model of the density of individuals *x* of continuous age *b* in an organism with a single life stage can be written as

(1) $$x_{i+1}\left(b\right)=\int_{\alpha }^{\gamma }x_{i}\left(a\right)k\left(b,a\right)da,$$

in which *x*_*i*_(*a*) is the density of individuals of age *a* at time step *i*,* k*(*b*,* a*) is the projection kernel that determines growth, mortality, and aging as the organism progresses from age *a* to age *b*. The bounds on the integral (α and γ) are upper and lower age bounds (Ellner & Rees, 2006). In the special case where growth and aging do not depend on age, Equation (2) can be rewritten as a convolution integral:

(2) $$x_{i+1}\left(b\right)=\int_{\alpha }^{\gamma }x_{i}\left(a\right)k\left(b-a\right)da.$$

When using the convolution integral above, we assume that demography is independent of age within a particular life stage. Although the assumption that demography is independent of age within stage is tenable for many organisms with life cycles that are divided into numerous short‐duration life stages, it obviously represents a simplification of reality (Tuljapurkar & Horvitz, 2006; Tuljapurkar, Steiner, & Orzack, 2009). For many organisms with fecundity that changes as a function of age, the assumption of age‐independent demography will not hold. In contrast, for annual plants and univoltine insects, reproduction often happens only at the endpoints of a multistage life cycle and within‐stage demography in the Northern Hemisphere is often more strongly influenced by seasonal forcing than by age within stage.

#### 2.1.2 Rate summation and seasonal forcing

The standard approach to modeling maturation is to define a variable, often called physiological age (*a*), which accumulates over time at a rate (*r*). In constant environmental conditions, in which the development rate does not vary in time (*r* = *r*_0_), physiological age can be computed by integration of the rate equation

(3) $$a\left(t\right)=\int_{0}^{t}r_{0}ds.$$

This development equation underlies the advection term in the McKendrick–von Foerster equation (von Foerster, 1959; McKendrick, 1926). In natural environments, however, the development rate depends on environmental conditions (*E*) and environmental conditions typically vary with time. If environmental conditions have been recorded over time, they can be represented using an empirical time dependency (*E*(*t*)) and Equation (3) must be redefined:

(4) $$a\left(t\right)=\int_{0}^{t}r\left[E\left(s\right)\right]ds.$$

Due to this environmental variation in time, Equation (4) cannot generally be computed analytically and must instead be numerically integrated by breaking the time interval (*t*) into a number (*n*) of small increments. Moreover, as environmental conditions are usually censused at discrete times (*t*_*i*_) separated by regular intervals (Δ*t*), Equation (4) can be approximated using a rate summation model (Logan, 1988):

(5) $$a\left(t_{n}\right)\approx \sum_{i=1}^{n}r\left[E\left(t_{i}\right)\right]\Delta t,$$

which is a Riemann sum (from basic integral calculus) approximation of Equation (4). This rate summation (Equation (5)) forms the basis of the aging process in variable environments.

To complete the age‐structured model (in the absence of mortality), the rate function in Equation (5) must be defined. Poikilotherm development rates depend on the temperature of their local environment (Sharpe & DeMichele, 1977) and so *E*(*t*_*i*_) in Equation (5) is replaced with *T*(*t*_*i*_). Many options exist for temperature‐dependent development rate models. Whereas rate models based on degree days assume that development accrues at a rate that is a linear function of temperature above a minimum temperature threshold (Dennis et al., 1986; Kemp et al., 1986; Pruess, 1983), more realistic rate models account for nonlinear temperature dependence (Régnière, Powell, et al., 2012; Schoolfield et al., 1981; Sharpe & DeMichele, 1977). We do not endorse one model over another here as our aim is to provide a general framework that can accommodate any environment‐dependent rate function.

#### 2.1.3 Stochastic rate variation

Due to phenotypic variation, environmental stochasticity, or sampling effects, the development rate at any given temperature will vary among individuals or populations (Gilbert et al., 2004; Régnière, Powell, et al., 2012). To account for this rate variability, we assume that development rate of individual *j* is a random variable (*R*_*j*_). Thus, we write a stochastic version of the rate summation (Equation (5)):

(6) $$A_{j,n}=\sum_{i=1}^{n}\left(R_{j,i}\right)\Delta t,$$

where *A*_*j*,*n*_ is the random level of development (age) accumulated by individual *j* after time interval *n*. Note that randomness in *A*_*j*,*n*_ is due to randomness in *R*_*j*,*i*_. For what follows, it is helpful to define an iterative reformulation of Equation (6) for any time step *i* ∊ {1, 2, …, *n*}.

(7) $$A_{j,i+1}=A_{j,i}+\left(R_{j,i+1}\right)\Delta t.$$

If we consider an organism that has age zero when born and age γ when it dies, then age in this organism with a single life stage is bounded (*A*_*j*,*i*_ ∊ [0, γ]). Note that the sum of random variables can be computed by convolving the probability density functions of the random variables in the sum (Hogg & Craig, 2005). Thus, we can represent the outcome of the sum in Equation (7) using the following convolution

(8) $$x_{i+1}\left(b\right)=\int_{0}^{\gamma }x_{i}\left(a\right)k_{i}\left(b-a\right)da,$$

where *x*_*i*_(*a*) is the probability density function of individuals of age *a* in time step *i* and *k*_*i*_(*b* − *a*) is the probability density function of the random variable *R*_*i*+1_ multiplied by Δ*t*, which can be thought of as the probability of aging from age *a* to age *b* in the time interval. Note that the kernel (*k*_*i*_(*b* − *a*)) varies from one time step to the next due to environmental forcing of the stochastic aging rate (*R*_*i*+1_). For now, we will assume that both probability density functions are defined on a strictly positive domain. The reader will recognize that Equation (8) is an integral projection model (compare Equation (8) to Equations (1) and (2)). However, it differs slightly from the way that integral projection models are traditionally defined in that the kernel (*k*_*i*_(*b* − *a*)) must integrate to one on *a* ∊ [0, ∞) even though the distribution of ages in the integral projection model is bounded by 0 and γ.

**Figure 2:**
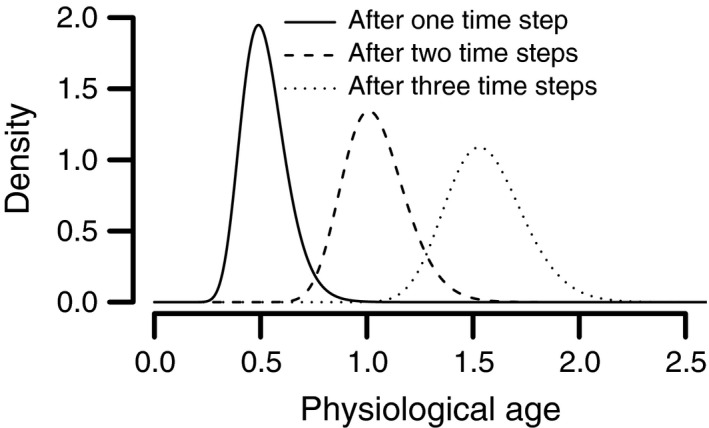
A demonstration of a stochastic age‐structured model where maturation is determined by a random development rate. The observed stochastic development rate in individual *j* in time step *i* is *R*_*j*,*i*_ is log‐normally distributed with a median of *r*_0_Δ*t* where the time step is equal to one unit time (Δ*t* = 1). Thus, the location parameter of the log‐normal distribution (μ) is equal to ln (*r*_0_Δ*t*), and σ is the scale parameter of the log‐normal distribution. The initial distribution of individuals at *t*_0_ was a Dirac delta function centered on the physiological of age of zero multiplied by 100 to represent 100 individuals that began with a physiological age of zero. The development rate parameter was *r*_0_ = 0.5(unit time)^−1^ and the scale parameter was σ = .2

If we assume that at *t*_0_ all individuals have zero accumulated development, then the initial condition can be written as

(9) $$x_{0}\left(a\right)=S_{0}\delta \left(a\right),$$

where *S*_0_ is a scalar that gives the population size and δ(*a*) is the Dirac delta function. The Dirac delta function (δ(*a*)) is a distribution that integrates to one and is zero everywhere except where *a* = 0. With this initial condition, the distribution of physiological age in the population will evolve over time as the stochastic aging process progresses. An example of the evolving distribution of physiological age for a hypothetical organism is given in Figure 2.

The stochastic integral projection model we derived in Equation (8) is an exact deterministic representation of the stochastic rate summation equation (Equations (6) and (7)), which is an approximation of continuous‐time nonautonomous differential equation models of seasonally forced phenology. The issue of the fidelity with which discretized models approximate their continuous counterparts is ubiquitous when computers are used to solve differential equations and relates back to the initial Riemann sum or Euler approximation of the solution of the underlying differential equation (Adler, 2005). Because the accuracy with which Equation (8) approximates the continuous‐time process will increase as the size of the time steps (Δ*t*) decreases, Equation (8) should enable arbitrarily exact approximation of the desired continuous‐time age‐structured model. To demonstrate that Equation (8) can represent the solutions of continuous‐time age‐structured models, we derive the extended von Foerster model (a continuous‐time age‐structured model) of Gilbert et al. (2004) starting from equations 8 and 9 in Appendix S1.

#### 2.1.4 Incorporating variable mortality

We have so far assumed no mortality, except due to old age, and modeled only the environment‐dependent aging process. Organisms, though, experience environment‐dependent mortality rates throughout their lives. Many insects (Lee, 1991) and plants (Inouye, 2000), for example, can be killed by extreme or aseasonal cold events. Adding mortality to the age‐structured model (Equation (8)) requires that we relax our initial restriction that *x*_*i*_(*a*) integrates to one on *a* ∊ [0, ∞) because, with mortality, the integral of *x*_*i*_(*a*) on *a* ∊ [0, ∞) will decrease over time (in the absence of birth) and can take on values less than one. Let *m*_*i*_ be the environment‐dependent probability of mortality in time step *i*. The age‐structured model with seasonally forced mortality then becomes

(10) $$x_{i+1}\left(b\right)=\left(1-m_{i}\right)\int_{0}^{\gamma }x_{i}\left(a\right)k_{i}\left(b-a\right)da.$$

This formulation assumes that mortality is not age‐dependent within a particular stage. In organisms with multiple stages, however, it is straightforward to construct models with stage‐specific mortality functions that are seasonally dependent. Adding density‐dependent mortality requires little added complexity as the only additional steps are defining an appropriate density‐dependent mortality function and computing the density of living individuals. Computing the total density of living individuals (*X*_*i*+1_) of any age within a particular life stage involves a second integration of Equation (10):

(11) $$X_{i+1}=\int_{0}^{\gamma }x_{i+1}\left(b\right)db.$$

The ease with which we can simultaneously accommodate environment‐dependent and density‐dependent mortality and environment‐dependent aging makes this approach more flexible than partial differential equation‐based models like the McKendrick–von Foerster equation.

#### 2.1.5 The stage and age‐structured model

**Figure 3:**
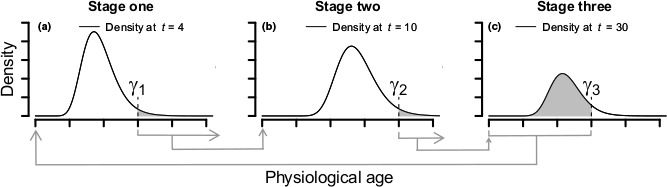
An illustration of a stage‐ and age‐structured model for a hypothetical organism with three life stages. In the first life stage (a), individuals that have passed the first developmental milestone (γ_1_) in each time step enter the second life stage at physiological age zero. In the second life stage (b), individuals that have passed the second developmental milestone, γ_2_, enter the third life stage at physiological age zero. In the third life stage (c), the third developmental milestone (γ_3_) corresponds to death due to old age. Thus, only individuals that have not yet reached that milestone are able to reproduce. Reproduction by individuals in the third life stage results in new individuals entering the first life stage at physiological age zero

To create a stage‐ and age‐structured model, we imagine that our model organism has *s* stages (where *s* is an integer). Each stage develops or ages according its own environment‐dependent development rate function *r*_*s*_[*E*(*t*_*i*_)]. The corresponding stochastic variable, *R*_*s*,*j*,*i*_, represents the stochastic development rate for individual *j* in time step *i* and stage *s*. When a critical level of development has been reached, individuals move to the first age in the next stage as shown in Figure 3. We represent the threshold level of development at which individuals in stage *s* develop into stage *s* + 1 with γ_*s*_. Consider an organism with three stages (such as egg, juvenile, and reproducing adult). We represent the distribution of ages within the three stages using *x*(*a*_*x*_), *y*(*a*_*y*_), and *z*(*a*_*z*_). These three distributions for age within stage represent distributions that result from the stochastic accumulation of physiological age in each of the stages represented by the random variables *A*_*j*,*i*,*x*_, *A*_*j*,*i*,*y*_, and *A*_*j*,*i*,*z*_ (that are analogous to *A*_*j*,*i*_ in Equation (7)). Using the same logic that underlies our derivation of Equation (8) from Equation (7), we extend the age‐structured integral projection model with variable mortality (Equation (10)) to form a stage‐ and age‐structured integral projection model of a hypothetical organism with three life stages:

(12a) $$x_{i+1}\left(b_{x}\right)=\left(1-m_{i,x}\right)\int_{0}^{\gamma_{1}}x_{i}\left(a_{x}\right)k_{i,x}\left(b_{x}-a_{x}\right)da_{x},$$

(12b) $$\begin{array}{cc} y_{i+1}\left(b_{y}\right) & =\delta \left(b_{y}\right)\left(\underset{\text{density that reached stage 2 by time}\;\;i+1}{\underbrace{\int_{\gamma_{1}}^{\infty }\int_{\gamma_{1}}^{\infty }x_{i}\left(a_{x}\right)k_{i,x}\left(b_{x}-a_{x}\right)\text{d}a_{x}\text{d}b_{x}}}-\underset{\text{density that reached stage 2 previously}}{\underbrace{\int_{\gamma_{1}}^{\infty }\int_{\gamma_{1}}^{\infty }x_{i-1}\left(a_{x}\right)k_{i-1,x}\left(b_{x}-a_{x}\right)\text{d}a_{x}\text{d}b_{x}}}\right)\\ & \;+\left(1-m_{i,y}\right)\int_{0}^{\gamma_{2}}y_{i}\left(a_{y}\right)k_{i,y}\left(b_{y}-a_{y}\right)\text{d}a_{y}, \end{array}$$

(12c) $$\begin{array}{cc} z_{i+1}\left(b_{z}\right) & =\delta \left(b_{z}\right)\left(\underset{\text{density that reached stage 3 by time}\;\;\;i+1}{\underbrace{\int_{\gamma_{2}}^{\infty }\int_{\gamma_{2}}^{\infty }y_{i}\left(a_{y}\right)k_{i,y}\left(b_{y}-a_{y}\right)\text{d}a_{y}\text{d}b_{y}}}-\underset{\text{density that reached stage 3 previously}}{\underbrace{\int_{\gamma_{2}}^{\infty }\int_{\gamma_{2}}^{\infty }y_{i-1}\left(a_{y}\right)k_{i-1,y}\left(b_{y}-a_{y}\right)\text{d}a_{y}\text{d}b_{y}}}\right)\\ & \;+\left(1-m_{i,z}\right)\int_{0}^{\gamma_{3}}z_{i}\left(a_{z}\right)k_{i,z}\left(b_{z}-a_{z}\right)\text{d}a_{z}. \end{array}$$

Note that each life stage in Equation (12a), (12b), (12c) has its own stage‐specific mortality function and aging kernel indexed by *x*,* y*, or *z*. In Equations 12b and 12c, we compute the individuals that have passed the γ_*s*_ threshold in the current time step by subtracting all of the individuals that exceeded the threshold by the previous time step from all of the individuals that exceeded the threshold by the current time step. We then multiply by the Dirac delta function to force all new individuals into the first age in the next stage. Note that in Equation (12a), (12b), (12c), we do not allow individuals to skip a life stage, so all individuals with physiological ages between γ_*s*_ and ∞ move to stage *s* + 1 in the next time step. For Equation (12a), (12b), (12c) to accurately represent demography, time steps need to be small enough to make the assumption of “no stage‐skipping” tenable.

The system in Equation (12a), (12b), (12c) provides the age distributions of individuals in each stage, but it does not give the population densities in each stage. A second integration of the elements of Equation (12a), (12b), (12c) is required to compute the total density of individuals in each stage as described in Equation (11). To complete the life cycle, we could add to Equation 12a an environment‐dependent birth function that describes the rate at which new individuals are born into the first stage as a function of the density of individuals in the reproductive stage (Figure 3).

### 2.2 Model application

#### 2.2.1 Temperature‐dependent mountain pine beetle biology

Typically adult mountain pine beetles fly from their brood trees in the summer or fall to attack new trees that, if successfully attacked, will become hosts for subsequent generation (Safranyik & Carroll, 2006). The phenology of the mountain pine beetle life cycle, including the timing of these summer flights, is strongly linked to temperature (Bentz, Logan, & Amman, 1991; Bentz & Powell, 2014; Logan & Bentz, 1999; Régnière et al., 2015; Régnière, Powell, et al., 2012). Flight phenology determines the success with which mountain pine beetle populations kill host trees (Logan & Powell, 2001; Powell & Bentz, 2009) because optimal tree killing and colonization by flying beetles rely on synchronous beetle flights (Logan, White, Bentz, & Powell, 1998) at a time that is seasonally adaptive for the initiation of new broods (Logan & Bentz, 1999; Logan & Powell, 2001). Adaptive seasonality results in the minimization of mortality in new broods due to lack of synchronization with seasonal fluctuations (Logan & Bentz, 1999; Logan & Powell, 2001).

To predict the phenology of the mountain pine beetle, researchers have parameterized development rate curves for each mountain pine beetle life stage based on the time required for stage completion when life stages are reared in the laboratory at various constant temperatures (Bentz et al., 1991). The curves that provide the most parsimonious fit to the data are nonlinear, hump‐shaped functions of temperature (Régnière, Powell, et al., 2012). The parameters of the current optimal rate curves for the egg, four larval instars, pupae and teneral adult life stages are given in Régnière, Powell et al. (2012) but are also reproduced in the appendices (Appendix S2). The full stage‐ and age‐structured phenology model can be constructed by linking age‐structured models such that individuals that finish development in one stage proceed to the next stage (see Section 3 for a more detailed description). The phenology model requires, as input, a time series of daily mean air temperatures in degrees centigrade that are then translated to under‐bark temperatures using a submodel (Bolstad, Bentz, & Logan, 1997).

Winter mortality is thought to be one of the main limiting factors of northward range expansion of mountain pine beetles (Bentz et al., 2010; Safranyik et al., 2010; Sambaraju et al., 2012) and is often cited as the cause of beetle population crashes that end outbreaks of mountain pine beetle infestation (Aukema et al., 2008; Sambaraju et al., 2012; Stahl, Moore, & McKendry, 2006). Because the egg, pupal, and teneral adult stages of the mountain pine beetle are less cold‐tolerant than the larval stages (Bentz & Mullins, 1999; Yuill, 1941), winter mortality in these stages is an important determinant of adaptive seasonality. However, modeling efforts that have attempted to incorporate both phenology and mortality have had mixed success (Dooley, Six, & Powell, 2015), largely because of uncertainty regarding the potential for the cold‐hardening process to interfere with the development rate process in the mountain pine beetle's larval stages (Dooley et al., 2015). Due to uncertainty regarding interaction between development and cold‐hardening processes, and because this interaction is the subject of ongoing research, we do not account for larval mortality in our mountain pine beetle phenology model even though mid‐winter larval mortality is possibly the most important mortality component in the mountain pine beetle life cycle (Safranyik & Carroll, 2006). We do, however, incorporate simple models for egg, pupal, teneral adult, and adult mortality using a temperature‐dependent step function in which the probability of mortality is zero when minimum temperatures are above −18°C and one when minimum temperatures are below −18°C (Régnière et al., 2015).

#### 2.2.2 Stochastic mountain pine beetle model

To test the ability of our deterministic integral projection models of phenology to capture the variability in stochastic systems, we developed a stochastic individual‐based model using the simulation approach of Régnière, Powell et al. (2012) to produce simulation results for comparison to those of the integral projection model. We simulated the maturation and mortality of a single mountain pine beetle brood, using a stochastic individual‐based approach in which each of the brood insects was separately simulated as an individual. The individual‐based simulation is forced using mean, minimum, and maximum daily air temperature data (°C) recorded between 15 July 2014 and 30 September 2015 at the Jasper warden station (Jasper, Alberta, Canada, Lat: 52.93, Lon: −118.03, Elevation: 1,020 m). The driving temperature data from Jasper are provided in the Supporting Information (Data S1). We initialized the stochastic model on 30 July 2014, with 82 eggs laid by a simulated mated female according to a temperature‐dependent oviposition model Régnière, Powell et al. (2012).

An assumption made during the conception and parameterization of the mountain pine beetle development rate models is that the rates are log‐normally distributed around the median development rate in the population (Régnière, Powell, et al., 2012). If the median development rate is given by the temperature‐dependent and stage‐dependent rate equation *r*_*s*_[*T*(*t*_*i*_)], then the location parameter of the log‐normal distribution (μ_*s*_) must be μ_*s*_ = ln (*r*_*s*_[*T*(*t*_*i*_)]). Thus, the probability density function on the random level of development accrued in one time step is

(13) $$p\left(r_{j};\mu_{s},\sigma_{s}^{2}\right)=\frac{1}{r_{j}\sigma_{s}\sqrt{2\pi }}\exp\left(\frac{{\left(\ln\left(r_{j}\right)-\ln\left(r_{s}\left[T\left(t_{i}\right)\right]\right)\right)}^{2}}{2\sigma_{s}^{2}}\right),$$

where $\sigma_{s}^{2}$ is the stage‐specific scale parameter of the log‐normal distribution that was also estimated in Régnière, Powell et al. (2012).

The algorithm for iterating the stochastic development model first computes the step and stage‐specific mean development rate *r*_*s*_[*T*(*t*_*i*_)]. Then, for each living individual, a random number is drawn from the log‐normal distribution $\text{LN}(\mu_{s}=\ln\left(r_{s}\left[T\left(t_{i}\right)\right]\right),\sigma_{s}^{2})$, and added to the level of development already accrued by the individual. After the development summation step, each individual is assigned to the appropriate life stage. Without loss of generality, we set γ_*s*_, the level of development required for maturation into the next life stage in all beetle life stages, to one (Régnière, Powell, et al., 2012). Thus, the individuals are moved among the life stages according to

$$s_{j}=\left\{\begin{array}{cc} s_{j} & \;\text{if}\;\sum_{1}^{i}\left(R_{s,j,i}\right)\Delta t\leq 1,\\ s_{j}+1 & \;\text{if}\;\sum_{1}^{i}\left(R_{s,j,i}\right)\Delta t>1. \end{array}\right.$$

The full simulation also requires a representation of mortality. We do not simulate larval mortality, but for the egg, pupae, teneral adult, and emerged adult life stages, the cold‐induced mortality model employs the step function described in the previous section.

We do not consider the mating process that leads to new life cycles but instead follow only a single life cycle by simulating up to the adult life stage. Computer code, written in R (R Core Team, 2016), that simulates the individual‐based model of the full life cycle is provided in the Supporting Information (Data S2).

#### 2.2.3 Integral projection mountain pine beetle model

Like in the stochastic mountain pine beetle model, the stage‐ and age‐structured integral projection model is forced using the Jasper mean daily temperature data and the time step and stage‐dependent median development rate is computed in each time step according to *r*_*s*_[*T*(*t*_*i*_)]. Instead of drawing a random number from the corresponding log‐normal distribution, the probability density function of the log‐normal distribution (Equation (13)) is convolved with the distribution of development already accumulated in the population as in Equation (8) to obtain an updated distribution on the level of development for each stage. Membership in each life stage as a result of development and mortality is then computed as described above. The mountain pine beetle‐specific equations are provided in Appendix S3. Computer code that simulates the stage‐ and age‐structured integral projection model of mountain pine beetle demography, written in R (R Core Team, 2016), is provided in the Supporting Information (Data S3).

#### 2.2.4 Cohort‐based mountain pine beetle model

To simulate a cohort‐based model of mountain pine beetle phenology and mortality for comparison with the analogous integral projection model, we reduced the age resolution at which we simulated the integral projection model of mountain pine beetle phenology and mortality such that within each life stage, individuals moved between 16 age cohorts before progressing to the next stage (compared to 128 age bins within each stage in the representation of the integral projection model in our code). We do not provide a separate script containing this code as it is essentially the same as the stage‐ and age‐structured integral projection model of mountain pine beetle demography and phenology already in the Supporting Information (Data S3).

#### 2.2.5 Model validation

To demonstrate the utility of the stage‐ and age‐structured integral projection model of mountain pine beetle phenology for prediction of phenological timing, we validated the model against mountain pine beetle flight trap data across Alberta, Canada. The model, as it is currently described, only simulates beetle development up to the adult stage and does not simulate flight. To simulate adult flight in our phenology model, we added a statement to the code in which individuals that had already developed into adults would fly if maximum daily temperatures exceeded 18.3°C, a flight temperature threshold based on observations of mountain pine beetle flight (McCambridge, 1971). Beetles are not able to fly indefinitely. To account for settling in beetles that expend their energy reserves for flight, we added an exponential settling function based on laboratory measurements of beetle flight propensity (Evenden, Whitehouse, & Sykes, 2014) and a simple model of mountain pine beetle dispersal (Goodsman et al., 2016).

Because we were unable to determine the exact time of adult mountain pine beetle attack and oviposition at specific locations or trees using data that were available to us, we used distributions of insect trap times from flight phenology data from the previous year to initialize our model at a variety of locations where we also had mountain pine beetle flight phenology data for the subsequent year, and where trees attacked by mountain pine beetle in the current year were confirmed. We then used BioSIM software (Régnière, Saint‐Amant, & Béchard, 2014) to spatially interpolate daily weather station data records of minimum and maximum temperatures for each location where we had confirmed mountain pine beetle attacked trees in the previous year. These temperature time series were used as input data for the model that we ran at each location. We calculated the cumulative number of simulated adult beetles that flying at each time in the flight window (usually between June and October).

In order to compare the simulated data to the normalized cumulative trap catch data for each region, we normalized the simulated data by dividing by the total number of simulated emerged adults to obtain a cumulative flight distribution of adults that was bounded from above by one. To test the quality of our phenological predictions and validate the integral projection model, we used a Kolmogorov–Smirnov test under the null hypothesis that the observed data and simulated data were similarly distributed. A failure to reject this null hypothesis corresponded to a positive validation result. The assumption that the number of trapped beetles at any time in the flight window is proportional to the density of flying beetles in the flight window is implicit in our model validation. Flight trap data for the locations and years used in this model validation are available in the Supporting Information (Data S4), and code written in R that simulates the predictions of the integral projection model with distributed start times is also available in the Supporting Information (Data S5).

## 3 RESULTS

**Figure 4:**
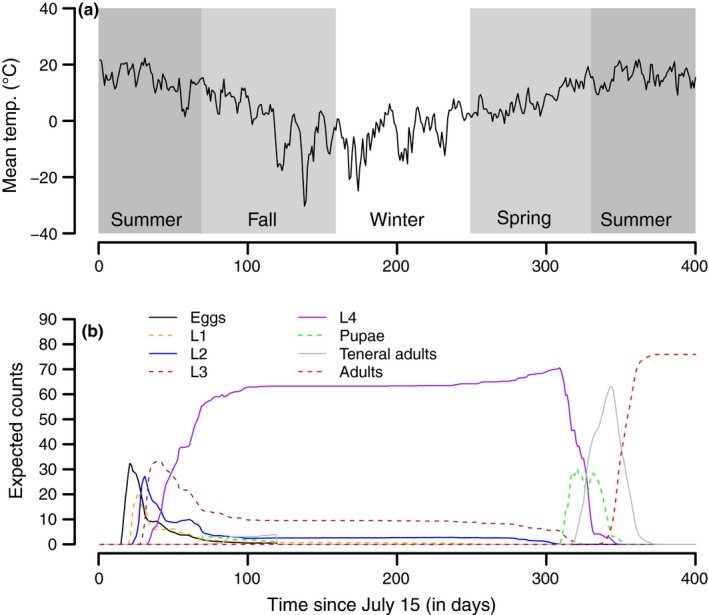
The predictions of the stage‐ and age‐structured integral projection model for temperature‐dependent development and mortality of a mountain pine beetle brood. The model is forced with (a) temperatures recorded at the Jasper warden station in Jasper, Alberta, Canada. The (b) expected number of individuals in each of the stages as determined by the stage‐ and age‐structured model. Note that the current version of the model does not account for larval mortality

**Figure 5:**
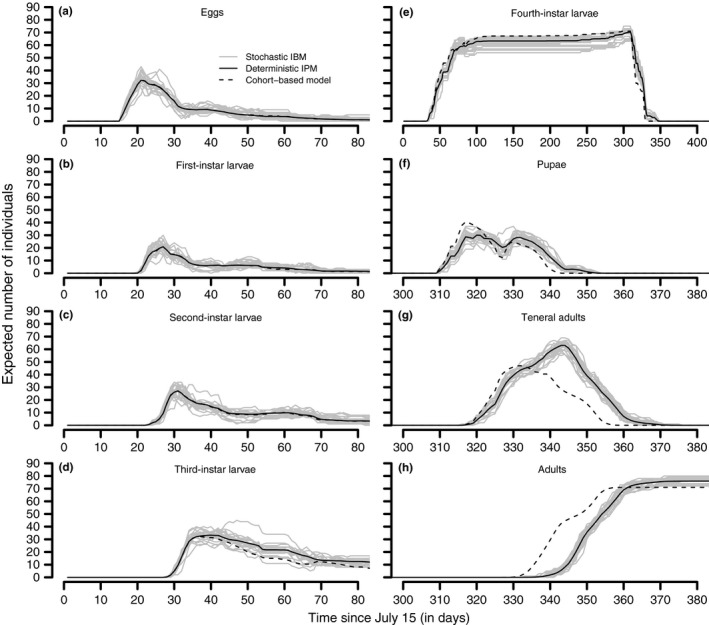
Comparisons of the predictions of the stage‐ and age‐structured integral projection model of mountain pine beetle phenology and mortality with the predictions of the stochastic age‐ and stage‐structured model on which it is based and a coarser cohort‐based model. For each mountain pine beetle life stage after oviposition (a–h), the predictions of the integral projection and cohort‐based models are overlaid on the trajectories predicted by 20 simulations of the stochastic model

Our results demonstrate the utility of our modeling approach by illustrating its accommodation of phenotypic variation in maturation rates in a model of mountain pine beetle phenology. The mountain pine beetle stage‐ and age‐structured model realistically simulated complex dynamics of temperature‐dependent mountain pine beetle phenology in a fluctuating environment (Figure 4). Although the integral projection model of mountain pine beetle dynamics is deterministic (mathematical details in Appendices S2 and S3), it captures the variability inherent in the stochastic model by correctly representing rate variation (Figure 5a–h). Due to the coarser nature of the cohort‐based model using 16 age cohorts within each life stage, some accuracy is lost relative to the underlying stochastic model (Figure 5a–h). Both the stochastic model and the integral projection model replicate the observed characteristics of mountain pine beetle demography well. In the early summer, for example, fourth‐instar larvae, pupae, and teneral adults can all be present at the same time (Figure 5b) and (Figure 5e–g).

The integral projection model of mountain pine beetle phenology and mortality is computationally faster than running Monte Carlo simulations of the stochastic model. Collectively, the 20 stochastic simulations shown in Figure 5 took approximately 170 times as long as the simulation of the corresponding integral projection model. Equivalently, each single run of the stochastic individual‐based model was approximately 8.5 times slower than running the integral projection model of mountain pine beetle phenology. Clearly, regardless of how many or how few Monte Carlo simulations are necessary to approximate the expected behavior of the stochastic system, the integral projection model is more computationally efficient than simulating the stochastic individual‐based model.

**Figure 6:**
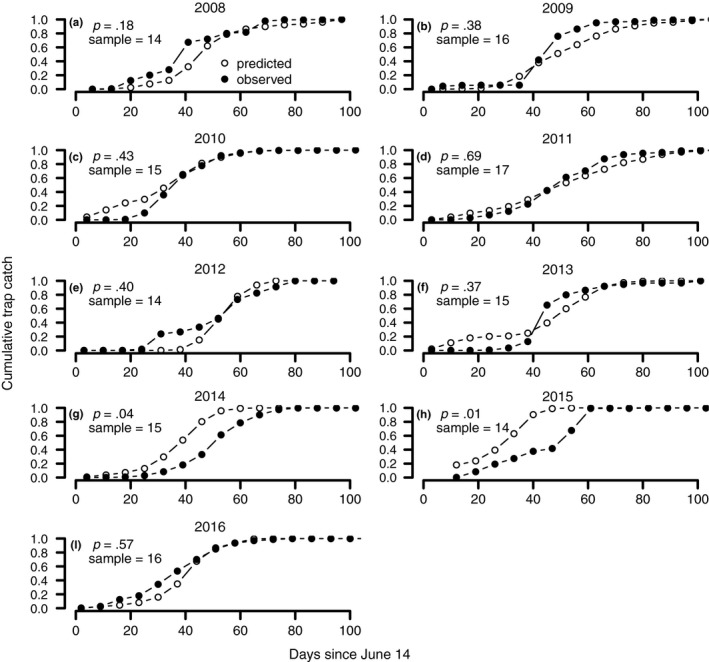
Validation plots comparing the predictions of adult flight time of the stage‐ and age‐structured integral projection model of mountain pine beetle phenology and mortality with observed trap catches of flying adult mountain pine beetles in Alberta, Canada from 2008 to 2016 (a–i). The cumulative trap catch curve is the normalized cumulative number of individuals caught. To test the quality of predictions, we used a Kolmogorov–Smirnov test and *p*‐values and sample sizes (not the number of insects but rather the number of records in the time series) for the tests in each year are given in the upper left corner. A *p*‐value larger than .05 means that we failed to reject the null hypothesis that the observed cumulative trap catch and predicted trap catch are similarly distributed (failing to reject this hypothesis corresponds to a positive validation result)

The validation of the integral projection model of mountain pine beetle phenology against flight trap data for mountain pine beetles shows that the model is able to predict mountain pine beetle flight phenology well over a variety of years and locations (Figure 6a–i). In each year, we tested the hypothesis that the observed data are distributed according to the predicted flight time distribution using a Kolmogorov–Smirnov test. In all years except 2014 and 2015, we failed to reject the hypothesis that the observed data are distributed according to the predicted data (Figure 6a–i). Our failure to reject this null hypothesis corresponds to a positive validation test result in every year but 2014 and 2015. Although we failed the rather strict validation test in 2014 and 2015, our prediction is shifted by only approximately 10 days relative to observations in 2014, and by 20 days in 2015. Despite the shifted mean or median flight times in 2014 and 2015, the shape of the predicted and observed cumulative catch curves are very similar in both years, indicating that the model correctly captured the variability in flight times if not the exact date of the mean flight time in those years.

## 4 DISCUSSION

We have developed a convolution‐based framework for constructing seasonally forced stage‐ and age‐structured integral projection models based on the rate summation concept. Rate summation models have been widely used to model the phenology of insects (Herms, 2004; Logan, 1988; Régnière, Powell, et al., 2012; Régnière, St‐Amant, et al., 2012) and plants (Ghersa & Holt, 1995; Osawa et al., 1983; Sharratt et al., 1989). Our process models use convolutions to compute random rate summations with natural variability in maturation rates, leading to age‐structured integral projection models that can be incorporated into stage‐structured models to form stage‐ and age‐structured models. Although the distribution that results from summing stochastic rates is not always analytically available, the use of fast Fourier transforms (Singleton, 1969) to numerically compute the outcomes of convolutions enables the framework to assume any imaginable distribution that describes rate variability. The convolution‐based integral projection modeling framework we propose differs from previous stochastic integral projection models (Rees & Ellner, 2009; de Valpine, 2009) in that our derivation is based on the rate summation framework, a widely used and accepted method for simulating phenology (Ghersa & Holt, 1995; Herms, 2004; Logan, 1988; Osawa et al., 1983; Régnière, Powell, et al., 2012; Régnière, St‐Amant, et al., 2012; Schoolfield et al., 1981; Sharpe & DeMichele, 1977; Sharratt et al., 1989; Wagner et al., 1984).

Our convolution‐based integral projection modeling framework demonstrates the connections between widely used phenology models based on degree days or on nonlinear rate functions and integral projection models that have become popular in ecology (Ellner & Rees, 2006; Rees & Ellner, 2009). The connection between these previously disparate model types is especially evident when models are derived for demography in constant environments. In constant environments or when development is modeled as a linear function of degree days, continuous‐time age‐ and stage‐structured models can be derived analytically using our convolution‐based framework. Thus, we have made explicit the relationship between extended von Foerster equations (Gilbert et al., 2004), the stochastic phenology models proposed by Dennis et al. (1986), and age‐structured integral projection models.

Although mathematical derivations are convenient in constant environments, we developed convolution‐based integral projection models for modeling environmentally forced dynamics. Rates that change from one time to the next due to a time‐varying environment can readily be incorporated in integral projection models. The mountain pine beetle system is ideal for demonstrating this capability of stage‐ and age‐structured integral projection models as researchers have developed mechanistic temperature‐dependent models of mountain pine beetle phenology and winter mortality. Using simulations of mountain pine beetle phenology and mortality in a fluctuating environment, we demonstrated that stage‐ and age‐structured integral projection models capture the effects of variability and can accommodate complex demographic submodels.

de Valpine (2009) developed stage‐ and age‐structured integral projection models assuming stochastic development, but the motivation behind the derivation of the integral projection models of de Valpine (2009) is fundamentally different from our own. de Valpine (2009) derived an integral projection model by integrating over continuous stochastic stage duration, while assuming that age is a discrete variable determined by time since birth (de Valpine, 2009). Conversely, we derived our integral projection model based on the convolution of continuous probability density functions that represent the stochastic variation of continuous physiological age. The probabilistic functions of de Valpine (2009) describe the probabilities of reaching particular age within a stage before transitioning to the next stage (stage duration) while the probability density functions in our integral projection models explicitly describe rate variability. Although development rates and stage duration are evidently linked, we can think of no simple way of accommodating seasonal forcing within models that assume an a priori distribution on stage duration. Accounting for seasonal forcing in models involving stage duration is difficult because stage duration changes as a function of environmental conditions that vary from one time step to a next in many organisms (e.g., poikilotherms in the Northern Hemisphere).

Integral projection models are a popular modeling approach in ecology with an extensive collection of literature that describes their underlying theory, application, and extensions (Coulson & Tuljapurkar, 2008; Ellner & Rees, 2006, 2007; Plard et al., 2016; Rees & Ellner, 2009; de Valpine, 2009). A significant proportion of the integral projection modeling literature focuses on stochastically derived integral projection models (Ellner & Rees, 2007; Rees & Ellner, 2009; de Valpine, 2009). In contrast to other stochastic integral projection models, our convolution‐based approach is derived starting from the rate summation idea. Therefore, parameters estimated for cohort‐based models (Focks et al., 1993, 1995; Logan, 1988) or individual‐based phenology and demography models (Régnière & Powell, 2013; Régnière, Powell, et al., 2012) based on the rate summation concept need not be estimated again for our modeling framework, but can rather be used directly in analogous convolution‐based integral projection models. Thus, many historic seasonally forced phenology models developed for a wide variety of organisms can be reformulated as integral projection models relatively easily using our framework.

A key concept that enabled us to model time‐evolving age structure as a distributed variable in our integral projection models was the separation of physiological age from time since birth. This distinction further differentiates our integral projection models from those of de Valpine (2009). Without the distinction between physiological age and time since birth, it would not make sense to model age as a distributed variable in the absence of variation in birth time. Randomly distributed development rates lead naturally to distributed levels of maturity in populations of individuals born at the same time.

Individual‐based models have been used to represent environment‐dependent demography under the influence of global climate warming for forest insects (Régnière & Powell, 2013; Régnière et al., 2015), but even with modern supercomputers, the computational burden and logistical complexity involved in simulating billions of individuals remain restrictive (Régnière et al., 2015). In this regard, integral projection and cohort‐based models have an advantage over individual‐based models; simulating a million individuals is no more computationally taxing than simulating a single individual. In theory, one can obtain the integral projection model in the limit as the number of cohorts in the cohort‐based model approaches infinity and cohort size approaches zero provided that the cohort‐based model includes a representation of development rate variability. Simulating integral projection models on a computer, however, requires discretization of distributions, which means essentially reverting them back to cohort‐based models. In spite of this reversion, a key advantage of our integral projection approach is that, due to its use of efficient mathematically based fast Fourier transforms (Singleton, 1969), implementing more accurate integral projection models rather than coarser cohort‐based models involves minimal increases in computational cost.

Due to their computational efficiency and scalability, convolution‐based integral projection models will enable researchers to surmount computational hurdles while maintaining the effects of stochastic variability and seasonal forcing. Moreover, convolution‐based integral projection models are amenable to incorporation directly into global climate models, enabling two‐way dependencies and interactions with the vegetation components of climate models and prediction of insect–vegetation dynamics under climate change.

## AUTHOR CONTRIBUTIONS

DWG derived the integral projection model from the stochastic rate summation model, wrote the initial draft of this manuscript, wrote the affiliated code, and performed the model validation. In addition to helping write this manuscript, the coauthors made the following contributions: BHA provided biological insight into the mountain pine beetle system; NM and RSM helped restructure the manuscript for a general audience; CX provided advice on the logical layout of the manuscript.

## CONFLICT OF INTEREST

None declared.
