# Supplementary material for: Incorporating variability in simulations of seasonally forced phenology using integral projection models
Source: Ecol Evol. 2017 Nov 26;8(1):162–75. doi: 10.1002/ece3.3590 (PMC5756895; doi:10.1002/ece3.3590)
Supplement: Supplementary file 1 [file ECE3-8-162-s001.pdf]

Appendix S1: Extended von Foerster Derivation

To demonstrate the connection between eqn 8 and classical age-structured models, we start with eqn 8, and derive an analytic solution for the extended von Foerster model (Gilbert *et al.*, 2004). The extended von Foerster model extends the classical McKendrick-von Foerster model (McKendrick, 1926; von Foerster, 1959) by allowing the aging rate to vary in the population (Gilbert *et al.*, 2004). Gilbert *et al.* (2004) incorporated normally distributed variation of development rate which requires the relaxation of our positive domain restriction on the probability density functions for the development rate and accumulated development. The normal probability density function with a mean given by r_0, the constant development rate in a constant temperature environment, is

$$p(r; r_0, \sigma^2) = \frac{1}{\sqrt{2\pi\sigma^2}}\exp\left(\frac{(r - r_0)^2}{2\sigma^2}\right). \qquad \text{(eqn A1.1)}$$

Although normally distributed rate variability permits biologically unrealistic negative development, for many combinations of rate and variability, the proportion of the population that develops negatively is negligible (Gilbert *et al.*, 2004).

Due to the scaling property of the normal distribution, the equivalent normal probability density function for a discretized time domain is

$$p(r; r_0\Delta\mathrm{t}, \Delta\mathrm{t}^2\sigma^2) = \frac{1}{\sqrt{2\pi(\Delta\mathrm{t})^2\sigma^2}}\exp\left(\frac{(r - r_0\Delta\mathrm{t})^2}{2(\Delta\mathrm{t})^2\sigma^2}\right), \qquad \text{(eqn A1.2)}$$

where Δt is the time increment.

If we start with the initial condition (eqn 9) and iterate eqn 8 for one time step using eqn A1.1 for $k(\cdot)$, we obtain

$$n_1(a) = (So)p(r; r_0\Delta\mathrm{t}, \Delta\mathrm{t}^2\sigma^2). \qquad \text{(eqn A1.2)}$$

Note, that we integrate eqn 8 over all reals instead of only over the positive reals as originally defined. By iteration of eqn 8 and by mathematical induction, we can find an analytic solution for n time steps when $S_0 = 1$:

$$x_n(a) = \frac{1}{\sqrt{2\pi\sigma^2 n(\Delta\mathrm{t})^2}}\exp\left(\frac{(a - r_0 n\Delta\mathrm{t})^2}{2\sigma^2 n(\Delta\mathrm{t})^2}\right). \qquad \text{(eqn A1.3)}$$

Recall that $t = n\Delta\mathrm{t}$. If we set $\Delta\mathrm{t} = 1$, we obtain

$$x(a,t) = \frac{1}{\sqrt{2\pi\sigma^2 t}}\exp\left(\frac{(a - r_0 t)^2}{2\sigma^2 t}\right), \qquad \text{(eqn A1.4)}$$

which is the analytic solution of the extended von Foerster model given in Gilbert *et al.* (2004). Similar analytic solutions can be obtained under the more realistic scenario where the probability distributions on the rate are defined strictly on the positive domain (for log-normally distributed rates, for example). These analytic solutions, however, are only valid for development at a constant rate because when the development rate varies over time, and the distribution is not stable, the convolution does not necessarily yield another probability density function of the same type.

References

Gilbert, E., Powell, J.A., Logan, J.A. & Bentz, B.J. (2004) Comparison of three models predicting developmental milestones given environmental and individual variation. *Bulletin of Mathematical Biology*, **66**, 1821–1850.

McKendrick, A.G. (1926) Applications of mathematics to medical problems. *Proceedings of the Edinburgh Mathematical Society*, **40**, 98–130.

von Foerster, H. (1959) Some remarks on changing populations. F.J. Stohlman, ed., *The Kinetics of Cellular Proliferation*. Grune & Stratton, New York.
