# Supplementary material for: Incorporating variability in simulations of seasonally forced phenology using integral projection models
Source: Ecol Evol. 2017 Nov 26;8(1):162–75. doi: 10.1002/ece3.3590 (PMC5756895; doi:10.1002/ece3.3590)
Supplement: Supplementary file 2 [file ECE3-8-162-s002.pdf]

Appendix S2: mountain pine beetle development rates

The rate equation for mountain pine beetle development, as defined in Régnière *et al.* (2012), is

$$r[T] = \psi\Bigg[\exp(\omega(T - T_b)) - \left(\frac{T_m - T}{T_m - T_b}\right)\exp(-\omega(T - T_b)/\Delta\mathrm{b}) - \left(\frac{T - T_b}{T_m - T_b}\right)\exp(\omega(T_m - T_b) - (T_m - T)/\Delta\mathrm{m})\Bigg], \quad \text{(eqn A2.1)}$$

where T is temperature (°C) and the six parameters (ψ, ω, T_m, T_b, Δb, Δm) vary between life stages. The development rate is given by eqn A3.1 except when $T \geq T_m$ or when $T \leq T_b$. When $T \geq T_m$ or when $T \leq T_b$, $r[T] = 0$. The parameter definitions and units are given in Table A2.1 and the estimated parameter values for each life stage are given in Table A2.2. An additional stage-specific parameter, σ_s, is estimated under the assumption that development rates in the population are log-normally distributed around the median given by eqn A2.1 as described in equation 13.

Table A2.1: Parameter definitions and units for eqn A2.1 developed by Régnière *et al.* (2012).

parameter	definition	units
T_b	base temperature for development	°C
Δb	width of lower thermal boundary layer	°C
T_m	maximum temperature for development	°C
Δm	width of upper thermal boundary layer	°C
ω	low temperature acceleration rate	$(°C)^{-1}$
ψ	peak rate control parameter	$(\text{day})^{-1}$

Table A2.2: Mountain pine beetle development rate parameter values estimated by Régnière *et al.* (2012). Note that σ corresponds to σ_ϵ in Table 4 and σ_δ in Table 5 of Régnière *et al.* (2012). Due to extreme sensitivity of the shape of the curve to parameter values, we have included many decimal places as recommended by Jacques Régnière (personal communication).

parameter	oviposition	egg	L1,	L2	L3	L4,	pupae	teneral
T_b	4.6341	7.0000	3.5559	6.9598	6.8462	16.2464	5.6300	4.2400
Δb	0.1	0.01930	0.10	0.09709	0.10	0.03905	0.10989	0.09997
T_m	27.7587	30.0928	29.2647	28.9047	28.7013	28.0000	28.5500	35.0000
Δm	3.0759	4.4175	3.8227	3.0374	2.5359	4.5504	2.8600	7.1479
ω	0.3684	0.2563	0.2398	0.3714	0.4399	0.2593	0.1532	0.1463
ψ	0.005199	0.02317	0.01082	0.01072	0.003892	0.05034	0.02054	0.01173
σ	0.2458	0.1799	0.2911	0.3799	0.3868	0.3932	0.2998	0.5284

References

Régnière, J., Powell, J., Bentz, B. & Nealis, V. (2012) Effects of temperature on development, survival and reproduction of insects: experimental design, data analysis and modeling. *Journal of Insect Physiology*, **58**, 634–647.
