# Supplementary material for: Incorporating variability in simulations of seasonally forced phenology using integral projection models
Source: Ecol Evol. 2017 Nov 26;8(1):162–75. doi: 10.1002/ece3.3590 (PMC5756895; doi:10.1002/ece3.3590)
Supplement: Supplementary file 3 [file ECE3-8-162-s003.pdf]

Appendix S3: Mountain pine beetle integral projection model

Here we describe the mathematics underlying a stage and age-structured integral projection model of seasonally forced mountain pine beetle demography. The model simulates the insect's progression through nine distinct life stages: oviposition, egg, four larval instars (L1, L2, L3, L4), the pupal, teneral adult, and adult stages. The ages within each of these stages are represented with a_q, a_r, a_s, a_t, a_u, a_v, a_w, a_x, and a_y (b_q, b_r, b_s, b_t, b_u, b_v, b_w, b_x, and b_y) respectively. The distributions of individuals of each age within each stage are are represented with $q(a_q)$, $r(a_r)$, $s(a_s)$, $t(a_t)$, $u(a_u)$, $v(a_v)$, $w(a_w)$, $x(a_x)$, and $y(a_y)$. As described in the materials and methods section and in Appendix S2, each stage has it's own temperature-dependent rate function which varies from one time step to the next and so the aging kernel $k_{i,s}(b-a)$ is indexed to show that it varies by life stage and by time step. In addition, the egg, pupa, teneral adult and adult life stages have mortality functions ($m_{i,s}$) that are step functions with 100% mortality happening at temperatures at or below -18°C and zero percent mortality otherwise. To obtain estimates of population densities, we perform a second integration for each integral projection model as shown in eqn 11 to obtain population densities for each life stage (Q_{i+1}, R_{i+1}, S_{i+1}, T_{i+1}, U_{i+1}, V_{i+1}, W_{i+1}, X_{i+1}, and Y_{i+1}).

The stage and age structured model for the oviposition, egg and first larval instar stages is

$$Q_{i+1} = (1 - m_{i,q}) \underbrace{Q_i \exp(-r_q[T_i]\Delta T)}_{\text{eggs not yet laid}}, \qquad \text{(eqn A4.1a)}$$

$$R_{i+1} = \underbrace{(1 - m_{i,q}) Q_i (1 - \exp(-r_q(T_i)\Delta T))}_{\text{eggs laid by time } i+1} +$$
$$(1 - m_{i,r}) \int_0^{\gamma_1} \int_0^{\gamma_1} r_i(a_r) k_{i,r}(b_r - a_r) \mathrm{d}a_r \mathrm{d}b_r, \qquad \text{(eqn A4.1b)}$$

$$S_{i+1} = (1 - m_{i,r}) \Bigg(\underbrace{\int_{\gamma_1}^{\infty} \int_{\gamma_1}^{\infty} r_i(a_r) k_{i,r}(b_r - a_r) \mathrm{d}a_r \mathrm{d}b_r}_{\text{L1 developed by time } i+1} -$$
$$\underbrace{\int_{\gamma_1}^{\infty} \int_{\gamma_1}^{\infty} r_{i-1}(a_r) k_{i-1,r}(b_r - a_r) \mathrm{d}a_r \mathrm{d}b_r}_{\text{L1 developed previously}} \Bigg) +$$
$$\underbrace{\int_0^{\gamma_2} \int_0^{\gamma_2} s_i(a_s) k_{i,s}(b_s - a_s) \mathrm{d}a_s \mathrm{d}b_s}_{\text{remained as L1 at time } i+1} . \qquad \text{(eqn A4.1c)}$$

Note that the method of simulation for the oviposition stage differs from all of the other stages in that we simulate the discretized differential equation $dQ/dR = -r_q[T_i]Q$ as described in Régnière *et al.* (2012) with an initial condition in each time step given by $Q(t_i) = Q_i$, where $\Delta T = t - t_i$.

The equations governing the dynamics of the second through the fourth instar larval stages, are

$$T_{i+1} = \Bigg(\underbrace{\int_{\gamma_2}^{\infty} \int_{\gamma_2}^{\infty} s_i(a_s) k_{i,s}(b_s - a_s) \mathrm{d}a_s \mathrm{d}b_s}_{\text{L2 developed by time } i+1} - \underbrace{\int_{\gamma_2}^{\infty} \int_{\gamma_2}^{\infty} s_{i-1}(a_s) k_{i-1,s}(b_s - a_s) \mathrm{d}a_s \mathrm{d}b_s}_{\text{L2 developed previously}} \Bigg) + \int_{0}^{\gamma_3} \int_{0}^{\gamma_3} t_i(a_t) k_{i,t}(b_t - a_t) \mathrm{d}a_t \mathrm{d}b_t, \qquad \text{(eqn A4.1d)}$$

$$U_{i+1} = \Bigg(\underbrace{\int_{\gamma_3}^{\infty} \int_{\gamma_3}^{\infty} t_i(a_t) k_{i,t}(b_t - a_t) \mathrm{d}a_t \mathrm{d}b_t}_{\text{L3 developed by time } i+1} - \underbrace{\int_{\gamma_3}^{\infty} \int_{\gamma_3}^{\infty} t_{i-1}(a_t) k_{i-1,t}(b_t - a_t) \mathrm{d}a_t \mathrm{d}b_t}_{\text{L3 developed previously}} \Bigg) + \int_{0}^{\gamma_4} \int_{0}^{\gamma_4} u_i(a_u) k_{i,u}(b_u - a_u) \mathrm{d}a_u \mathrm{d}b_u, \qquad \text{(eqn A4.1e)}$$

$$V_{i+1} = \Bigg(\underbrace{\int_{\gamma_4}^{\infty} \int_{\gamma_4}^{\infty} u_i(a_u) k_{i,u}(b_u - a_u) \mathrm{d}a_u \mathrm{d}b_u}_{\text{L4 developed by time } i+1} - \underbrace{\int_{\gamma_4}^{\infty} \int_{\gamma_4}^{\infty} u_{i-1}(a_u) k_{i-1,u}(b_u - a_u) \mathrm{d}a_u \mathrm{d}b_u}_{\text{L4 developed previously}} \Bigg) + \int_{0}^{\gamma_5} \int_{0}^{\gamma_5} v_i(a_v) k_{i,v}(b_v - a_b) \mathrm{d}a_v \mathrm{d}b_v. \qquad \text{(eqn A4.1f)}$$

The equations governing the dynamics of the pupal, teneral adult and adult stages, are defined by

$$W_{i+1} = \Bigg(\underbrace{\int_{\gamma_5}^{\infty} \int_{\gamma_5}^{\infty} v_i(a_v) k_{i,v}(b_v - a_v) \mathrm{da}_v \mathrm{db}_v -}_{\text{Pupae developed by } i+1}$$

$$\underbrace{\int_{\gamma_5}^{\infty} \int_{\gamma_5}^{\infty} v_{i-1}(a_v) k_{i-1,v}(b_v - a_v) \mathrm{da}_v \mathrm{db}_v}_{\text{pupae developed previously}} \Bigg) +$$

$$(1 - m_{i,w}) \int_0^{\gamma_6} \int_0^{\gamma_6} w_i(a_w) k_{i,w}(b_w - a_w) \mathrm{da}_w \mathrm{db}_w, \qquad \text{(eqn A4.1g)}$$

$$X_{i+1} = (1 - m_{i,w}) \Bigg(\underbrace{\int_{\gamma_6}^{\infty} \int_{\gamma_6}^{\infty} w_i(a_w) k_{i,w}(b_w - a_w) \mathrm{da}_w \mathrm{db}_w -}_{\text{tenerals developed by time } i+1}$$

$$\underbrace{\int_{\gamma_6}^{\infty} \int_{\gamma_6}^{\infty} w_{i-1}(a_w) k_{i-1,w}(b_w - a_w) \mathrm{da}_w \mathrm{db}_w}_{\text{tenerals developed previously}} \Bigg) +$$

$$(1 - m_{i,x}) \int_0^{\gamma_7} \int_0^{\gamma_7} x_i(a_x) k_{i,x}(b_x - a_x) \mathrm{da}_x \mathrm{db}_x, \qquad \text{(eqn A4.1h)}$$

$$Y_{i+1} = (1 - m_{i,y}) \underbrace{\int_{\gamma_7}^{\infty} \int_{\gamma_7}^{\infty} x_i(a_x) k_{i,x}(b_x - a_x) \mathrm{da}_x \mathrm{db}_x}_{\text{adults developed by time } i+1}. \qquad \text{(eqn A4.1i)}$$

Note that we do not account for the distribution of development in the adult stage but rather integrate the cumulative density of individuals that reach the adult stage Y_{i+1}.

References

Régnière, J., Powell, J., Bentz, B. & Nealis, V. (2012) Effects of temperature on development, survival and reproduction of insects: experimental design, data analysis and modeling. *Journal of Insect Physiology*, **58**, 634–647.
